# The long-term effects of perceived instructional leadership on teachers’ psychological well-being during COVID-19

**DOI:** 10.1371/journal.pone.0305494

**Published:** 2024-08-19

**Authors:** Xiu-Mei Chen, Xiao Ling Liao, I-Hua Chen, Jeffrey H. Gamble, Xing-Yong Jiang, Xu-Dong Li, Cun-Xu Bo

**Affiliations:** 1 School of Information Engineering, Shandong Youth University of Political Science, Jinan, Shandong, China; 2 Faculty of Education, Qufu Normal University, Qufu, Shandong, China; 3 Faculty of Education, Jiangxi Science and Technology Normal University, Nanchang, Jiangxi, China; 4 Chinese Academy of Education Big Data, Qufu Normal University, Qufu, Shandong, China; 5 Department of English, National Changhua University of Education, Changhua, Taiwan; 6 Yangan Primary School of Qionglai City, Qionglai, Sichuan, China; 7 Gaogeng Nine-year School, Qionglai, Sichuan, China; 8 Shandong Provincial Institute of Education Sciences, Jinan, Shandong, China; St John’s University, UNITED STATES OF AMERICA

## Abstract

The COVID-19 outbreak led to widespread school closures and the shift to remote teaching, potentially resulting in lasting negative impacts on teachers’ psychological well-being due to increased workloads and a perceived lack of administrative support. Despite the significance of these challenges, few studies have delved into the long-term effects of perceived instructional leadership on teachers’ psychological health. To bridge this research gap, we utilized longitudinal data from 927 primary and secondary school teachers surveyed in two phases: Time 1 in mid-November 2021 and Time 2 in early January 2022. Using hierarchical linear modeling (HLM), our findings revealed that perceptions of instructional leadership, especially the "perceived school neglect of teaching autonomy" at Time 1 were positively correlated with burnout levels at Time 2. Additionally, burnout at Time 2 was positively associated with psychological distress and acted as a mediator between the "perceived school neglect of teaching autonomy" and psychological distress. In light of these findings, we recommend that schools prioritize teachers’ teaching autonomy and take proactive measures to mitigate burnout and psychological distress, aiming for the sustainable well-being of both teachers and students in the post-pandemic era.

## 1 Introduction

In response to the outbreak of COVID-19, countries worldwide implemented protective measures, such as physical distancing, to prevent the spread of the virus, resulting in the closure of schools globally [1]. The closure of schools has not only affected students’ psychological well-being [2–5], but has also caused a significant level of stress among teachers [6, 7]. Studies indicate that teachers experienced pressure during the closure period due to mandatory teaching of online courses [8], increased teaching workloads [9], lack of support from administrators [10, 11], and poor communication with students and parents [9]. Additionally, teachers suffered from symptoms such as anxiety, depression, and sleep disturbances [12]. As such, the literature has provided mounting evidence to suggest that COVID-19 has caused considerable psychological distress among teachers [13–15].

Recent studies have underscored the potential long-term consequences of pandemic-induced stress, which can erode protective factors such as teachers’ resilience. This erosion can lead to burnout [16] and adversely affect their psychological well-being [17–19]. The challenges are compounded by the fact that school closures and the shift to online teaching have heightened the risk of burnout among teachers [20]. This exacerbates their already significant levels of psychological distress [21, 22], leading researchers to delve deeper into the factors contributing to job burnout and psychological distress among educators.

Building on this, individual-level factors during COVID-19 have been extensively studied. These include role conflict [23], professional experience (such as the number of years spent teaching) [24], teacher professional identity (which encompasses individual beliefs, values, and commitments related to the teaching profession) [25], and perceptions about one’s ability to control situations [26]. This also covers competence in online teaching tasks [27] and anxiety related to communicating with parents [28]. On the organizational front, Maslach et al. [29] posited that burnout stems from extended exposure to work-related stressors. Thompson et al. [30] introduced the Six Areas of Worklife model, pinpointing workload, control, reward, and values as organization-level factors linked to burnout, especially during the COVID-19 era. Other organization-level factors contributing to teacher burnout include work climate, work pressure, perceptions of collective exhaustion among peers, disruptions to conventional classroom teaching [31], diminished administrative support [28, 32], and supervisory management styles [33, 34]. Research has also highlighted the correlation between principals’ leadership styles and teacher burnout [35–37]. Moreover, numerous studies have identified teacher burnout as a significant predictor of psychological distress in educators [21, 38, 39].

While the significance of both individual-level and organization-level factors related to burnout has been assessed in the context of COVID-19, organization-level factors have not been sufficiently evaluated. Indeed, the education department should place greater emphasis on factors at the organizational level when implementing decisive measures to address them. Instructional leadership, a pivotal aspect of school leadership [40, 41], has yet to be thoroughly explored in terms of its impact on teachers’ well-being during the pandemic. To date, there seems to be a gap in the literature regarding how teachers’ perceptions of instructional leadership influence their experiences of burnout and psychological distress, especially during school closures. This gap is particularly evident in studies focusing on the longitudinal effects of perceived instructional leadership on the mental health of Chinese teachers. Given this context, this study seeks to address the following research question: *How do teachers’ perceptions of instructional leadership affect their subsequent experiences of burnout and psychological distress*?

To address the above gap, our study undertook two waves of data collection: the first wave was gathered during the period of online teaching when campuses were closed, aiming to gauge teachers’ perceptions of instructional leadership. The second wave was collected after the resumption of face-to-face classes to assess teacher burnout and psychological distress. The objective of this paper is to explore the relationship between perceived instructional leadership and subsequent burnout and psychological distress using hierarchical linear modeling (HLM). In this context, teachers’ perceptions of instructional leadership are considered at the school level, while burnout and psychological distress are evaluated at the individual (teacher) level. The subsequent section will present the model and research hypotheses.

## 2 Model and hypothesis

**Fig 1:**
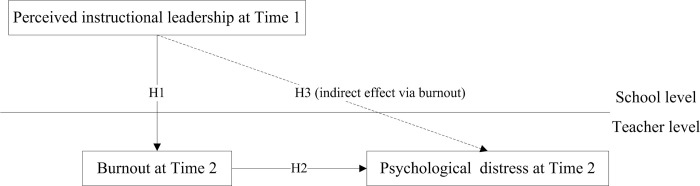
Research framework. The dotted line represents the indirect effect of perceived instructional leadership at Time 1 on psychological distress at Time 2.

In the present research, we employed longitudinal data to systematically examine the influence of teachers’ perceptions of instructional leadership on subsequent manifestations of job burnout and psychological distress, as delineated in Fig 1. To operationalize the construct of perceived instructional leadership, we grounded our categorization within the tenets of the Self-Determination Theory (SDT), segmenting it into three distinct categories. To elucidate the interrelationships among these variables, we anchored our investigation in the Stressor-Strain-Outcome (SSO) model, as proposed by Koeske and Koeske [42], subsequently formulating pertinent research hypotheses.

### 2.1 The SSO model

The Stressor-Strain-Outcome (SSO) model explains how work-related stressors negatively impact employee behavior through psychological strain, and conceptualizes strain as a mediating factor [42]. Stressors, in the SSO model, are environmental stimuli that employees perceive as bothersome and disruptive, such as excessive workload, a lack of support, and conflicting roles [42–44]. Strain, on the other hand, is a negative reaction to environmental stimuli that disrupts employees’ concentration, effecting their physiology and mood [42, 45], with burnout as a common manifestation [42, 43]. Outcome refers to the lasting behavioral or psychological effects of chronic stress and strain, such as physical or psychological symptoms (e.g., psychological distress in the workplace).

Based on the aforementioned concerns, three perceptions of school instructional leadership were evaluated by this study as disruptive environmental stimuli (i.e., stressors): perceived school neglect of teaching autonomy, perceived school neglect of teaching competence, and perceived school emphasis on competitive relationships during the COVID-19 pandemic.

Studies shown that burnout is often conceptualized as a strain in response to environmental stimuli in SSO model [42, 43]. The construct of job-related burnout was proposed by Freudenberger [46] to describe the extreme physical and emotional exhaustion experienced by individuals due to excessive workloads. Maslach et al. [29] later defined burnout as "a prolonged response to chronic emotional and interpersonal stressors on the job," characterized by exhaustion, cynicism, and inefficacy. Emotional exhaustion, in particular, is considered the central component of burnout [42, 47]. In the context of the COVID-19 pandemic, emotional exhaustion maybe a negative reaction to teachers’ perceived instructional leadership [48, 49].

As per the SSO model, stressors produced by three perceptions of instructional leadership may result in psychological distress (outcome) in teachers, with burnout (strain) mediating the relationship between the two. In the following subsections, the hypothesized relationships between these variables are presented sequentially.

### 2.2 Operationalizing perceived instructional leadership during the COVID-19 pandemic: A tripartite categorization based on SDT

Instructional leadership is widely recognized as the cornerstone of school leadership [40, 41]. Narrow conceptions of instructional leadership focus solely on teacher behaviors that augment student learning, whereas broader interpretations encompass issues related to both organizational and teacher culture [50]. According to Alig-Mielcarek and Hoy [51], instructional leadership comprises three primary components: (1) defining and communicating goals, (2) monitoring and providing feedback on the teaching and learning process, and (3) promoting and emphasizing the significance of professional development. Consequently, instructional leadership has emerged as an indispensable element of school reform and enhancement [51]. It influences a myriad of factors pivotal to the resilience of educational institutions, ranging from "organizational silence" (where crucial events or concerns remain unvoiced) to "organizational attractiveness" (reflecting positive sentiments towards an institution) [52].

The construct of instructional leadership in this study differs from the predominate perspective, which emphasizes leaders’ roles in stimulating teachers’ effectiveness in teaching and learning and improving students’ outcomes [53]. During the COVID-19 period, a more directive leadership style is indispensable to efficiently guide teachers in adapting to the unfamiliar task of online teaching [54, 55], and it can be considered a special form of "instructional leadership" under pandemic conditions. It is uniquely adapted for the pandemic context and can reflect the teachers’ perceived instructional leadership in the context of epidemic. Specifically, drawing from the SDT, this study categorizes teachers’ perceptions of instructional leadership during school closures into three distinct categories: perceived school neglect of teaching autonomy, perceived school neglect of teaching competence, and perceived school emphasis on competitive relationships. As posited by SDT, every individual harbors three fundamental psychological needs: autonomy, competence, and relatedness [56, 57]. The fulfillment of these needs is essential for an individual’s holistic development, and any deficiency can adversely impact their psychological well-being [57]. In this context, perceived school neglect of teaching autonomy denotes teachers’ sentiments that schools overlooked their online teaching autonomy, compelling them to adhere to specific teaching standards and methodologies, thereby affecting perceived autonomy. Perceived school neglect of teaching competence signifies teachers’ perceptions that schools disregarded their online teaching competence during the closure, marked by a lack of provision for necessary online teaching training and an apparent indifference to the challenges of online/distance teaching, thereby affecting perceived competence. The perception of the school emphasizing competitive relationships suggests environments where competition among teachers was unduly promoted, engendering a detrimental atmosphere concerning relatedness. These constructs, which pertain to the neglect of teacher autonomy and competence and the prioritization of competition over collaboration, can be considered stressors in pandemic context. They have largely remained unexplored empirically. In contrast, supportive instructional leadership styles, which have been linked with a sustainable sense of agency, teacher expertise, and positive peer relationships, are documented in sustainable education literature [58, 59].

### 2.3 Perceived instructional leadership and burnout

In this study, we examine the impact of perceived instructional leadership on teachers’ job burnout during the school closure period. In the previous research, it was found that principals’ leadership was related with teacher burnout [35–37]. Eyal and Roth [35] found that while transactional leadership (which seeks efficiency through monitoring and ensuring compliance through rewards and punishments) was positively correlated with burnout, transformational leadership (characterized by empowering and fostering individuals’ sense of mission through encouragement of innovation based on individual needs) was negatively correlated with burnout. Collie’s findings [36] highlighted that autonomy-supportive leadership (which refers to practices that promote individuals’ self-initiation and empowerment) was associated with lower emotion exhaustion and autonomy-thwarting leadership (which refers to practices that exert external control and reduce individuals’ self-determination) was positively associated with emotional exhaustion. Based on instructional leadership has been accepted as the core of school leadership [40, 41], our first hypothesis (Hypothesis 1) is that teachers’ perceived instructional leadership would be positively associated with teachers’ job burnout. Specifically, we propose three sub-hypotheses based on the dimensions of instructional leadership that have been suggested as significant stressors, based on the SSO.

H_1a_: Perceived instructional leadership that neglects teaching autonomy will have a positive relationship with job burnout. Previous research has shown a strong relationship between burnout and autonomy [29, 36, 60, 61]. Teachers who are unable to choose their own teaching methods during remote teaching may experience negative attitudes towards teaching activities, dissatisfaction with their work, and depression [62].

H_1b_: Perceived instructional leadership that neglects teaching competence will have a positive relationship with burnout. During the school closure period, teachers were not provided with required training for online teaching, and some may feel that the school was not paying attention to their teaching abilities. This lack of support may result in increased teaching pressures and a sense of incompetence, leading to burnout [34, 63].

H_1c_: Perceived instructional leadership that emphasizes competitive relationships will have a positive relationship with burnout. Instructional leadership that emphasizes competition among teachers may lead to a lack of feedback from colleagues and leaders during online instruction, which has been shown to contribute to burnout [64].

### 2.4 Burnout and psychological distress

Job burnout is a persistent, negative, and work-related psychological condition that can lead to turnover intention [65] (for example, among Chinese high school teachers during the pandemic), reduced productivity [66] (for example, among primary and secondary school teachers in English), and psychological distress such as anxiety and depression both in the general population [29, 67] and among schoolteachers [68]. Teachers belong to a profession that is more likely to experience work-related stressors and psychological distress than other occupations [69]. As a group at high risk of job burnout [70], teachers have drawn extensive attention from researchers [14, 71, 72]. Shin et al. [38] used a three-wave longitudinal data to show that burnout among Korean middle and high school teachers predicted subsequent depressive symptoms. Similarly, in a scoping review, Agyapong et al. [39] found that teacher burnout could provoke symptoms such as anxiety and depression.

Based on the above facts, we propose the second research hypothesis: teachers’ burnout will be positively associated with psychological distress (Hypothesis 2). This hypothesis suggests that the experience of burnout in teachers is likely to result in psychological distress, given the high prevalence of psychological distress among teachers and the evidence linking burnout to subsequent depressive symptoms and other negative mental health outcomes.

### 2.5 The mediation of burnout between perceived instructional leadership and psychological distress

According to the SSO model, job stress does not necessarily lead directly to specific outcomes but may act on outcomes through a mediating mechanism (in this case, burnout) [42]. This mediating effect of burnout has been documented in various studies. For instance, Koeske and Koeske [73] found that emotional exhaustion mediated stressful events experienced by students and their physical and mental health symptoms. In two other studies, Dhir et al. [74] and Pang [75] found that social media fatigue mediated excessive media use and anxiety and depression as well as perceived information overload and emotional stress and social anxiety.

The independent variables from the above literature [73–75], including stressful events experienced by students, excessive media use, perceived information overload, and the three types of teachers’ perceived instructional leadership assessed in this study, are all prominent stressors. The dependent variables, such as anxiety and depression, represent different forms of psychological distress. Therefore, we hypothesize that burnout may mediate the relationship between teachers’ perception of instructional leadership (neglect of teaching autonomy, neglect of teaching competence, and emphasis on competitive relationships) and psychological distress (Hypothesis 3).

## 3 Methods

### 3.1 Participants

In this study, participants were recruited from Shangrao City, Jiangxi Province, China. Due to the COVID-19 outbreak in the city during October 2021, face-to-face teaching was cancelled for the city’s primary and secondary schools by the municipal government, beginning on November 3, 2021. After a month of strict restrictions, the outbreak was brought under control, and the campus reopened for face-to-face instruction. During this period, we conducted an online survey, with the assistance of the city’s education department, comprised of two waves. The first wave was conducted to investigate teachers’ perceived instructional leadership during school closures (Time 1: mid-November 2021). The second wave of the study examined teachers’ burnout and psychological distress within 2 to 3 weeks of resuming face-to-face teaching (Time 2: early-January 2022).

A priori sample size estimation was conducted using the Optimal Design Software [76, 77]. With the support of the city’s education department, we were able to involve more than 100 schools in this survey. For the intended HLM analysis, given a cluster number of 100, a desired power of 0.8, an expected effect size of 0.30, and a significance level set at 5% (0.05), the a priori estimation yielded a requirement of five subjects per cluster (refer to S1 Fig). Based on this outcome, we deduced that for cluster numbers exceeding 100, having 5 subjects per cluster would be adequate. This conclusion aligns with findings from previous studies [78, 79]. These studies emphasized that to achieve adequate power, it’s more beneficial to increase the number of sampled clusters. Typically, sample sizes of up to 60 at the highest level and k+2 at the lower level (when there are k independent variables) are required.

This study was approved by the Jiangxi Association of Psychological Counselors (IRB ref: JXSXL-2020-J013), and with the assistance of the local education authority, data was collected via a hyperlink via convenience sampling. As participation was voluntary, participants were asked to include their email addresses if they wished to participate in a follow-up survey. There were 1,642 teachers who provided their email addresses and completed the longitudinal survey. To ensure data quality, we eliminated participants whose reported age was less than 18 and whose response time to all questions was less than 150 seconds. Additionally, we decided to exclude schools with participants of less than 4, considering the issue of representativeness and the required sample size [78, 79]. As a final sample, 103 schools and 927 primary and secondary teachers were included, with a minimum of five teachers per school.

### 3.2 Measures

Demographic variables such as gender, teaching experience, subject of instruction and school type (primary school or secondary school), were collected. At Time 1, participants were asked to rate their perception of instructional leadership in the context of mandatory online instruction. At Time 2, participants were asked to report their levels of burnout and psychological distress over the preceding two weeks. The following subsections provide a detailed description of the measurement tools used in this study, and the items of the questionnaires are listed (see S1–S3 Tables) in appendix.

#### 3.2.1 Perceived instructional leadership

To our knowledge, there isn’t a tool specifically designed to measure teachers’ perceptions of instructional leadership during periods of mandatory online teaching, such as those experienced during the pandemic. In the context of epidemic, a more directive leadership style is essential to guide teachers in the face of online teaching [54]. For the purpose of assessing teachers’ perceptions of this special form of instructional leadership at Time 1, we utilized the Psychological Need Thwarting Scale of Online Teaching (PNTSOT) developed by Yi et al. [80]. The alignment between perceived instructional leadership and the PNTSOT is illustrated in S2 Fig.

The PNTSOT was initially developed to assess the extent of psychological need thwarting during online teaching. In accordance with the CFA results in [80] (CFI = 0.966, NNFI = 0.955, RMSEA = 0.09, and SRMR = 0.05) and revised results in [72] (CFI, NNFI ranged from 0.960 to 0.999; RMSEA and SRMR were both less than 0.09), these results indicate that PNTSOT has ideal factorial validity among primary and secondary schoolteachers.

In this study, the three subscales of the PNTSOT (autonomy, competence and relatedness thwarting) were considered as direct reflections of perceived instructional leadership (perceived school neglect of teaching autonomy, perceived school neglect of teaching competence and emphasis on competitive relationships) by teachers. For each question, a seven-item Likert-type scale was used, ranging from "strongly disagree" to "strongly agree". The three variables for psychological need thwarting were aggregated into school-level variables which corresponded to the three types of perceived instructional leadership. Through HLM, high-level data can be derived from the aggregation of low-level data. To establish the plausibility of the aggregation, the values of within-group agreement (*r*_*wg*_) were calculated and they were found to have adequate consistency (*r*_*wg*_ values for perceived school neglect of teaching autonomy, neglect of teaching competence, and emphasis on competitive relationships were 0.77, 0.74 and 0.82). Values of *r*_*wg*_ between 0.70 and 0.79 indicated moderate agreement, and values of .80 and above indicated strong agreement [81]. As a result, it is was deemed reasonable to aggregate teacher-level data to school-level data and use them as independent variables for this study. The following will explain the correspondence between the three sub-dimensions of the PNTSOT and the three types of perceived instructional leadership.

Perceived school neglect of teaching autonomy refers to instructional leadership in which teachers felt that schools did not value their teaching autonomy and forced them to use specific teaching methods during online teaching. Perceived school neglect of teaching autonomy can be described by the autonomy thwarting subscale of the PNTSOT in terms of the following four items: “During online courses during the pandemic, I cannot decide for myself how I want to teach”, “During online teaching work during the pandemic, I feel there is pressure that affects my behavior and requires me to comply in a certain way”, “I have to follow a prescribed online teaching style during the pandemic” and “During the pandemic, I feel pressure from the external environment that limited me in choosing a particular online teaching style”. The higher the score, the more pronounced the perception of neglected teaching autonomy. Teachers perceptions of school neglecting teaching autonomy in this study demonstrated a high level of internal consistency (Cronbach’s α = 0.79, McDonald’s ω = 0.79).

Perceived school neglect of teaching competence means that teachers believed their schools did not provide necessary online teaching training. They also paid little attention to their online teaching during the school closure period. Teachers felt that they had few opportunities to acquire more online teaching experiences. This sense of neglect can be described through competence thwarting in PNTSOT. The four items of competence thwarting included “There are some online teaching situations that make me feel incapable in my daily work environment during the pandemic” and “Due to the lack of training opportunities in my environment, I feel that I am not capable of performing online teaching tasks”. As a result of these items, it appears that schools may be neglecting teachers’ online teaching ability. A higher score indicates a higher level of perceived neglect of teaching competence. There is an acceptable degree of internal consistency from our data (Cronbach’s alpha = 0.84, McDonald’s alpha = 0.86) for perceived school neglect of teaching competence.

Perceived school emphasis on competitive relationship refers to teachers’ belief that schools value teachers’ competition. This variable can be assessed using relatedness thwarting in the PNTSOT, in which the four items include “I feel disconnected from other colleagues and leaders when teaching online during the pandemic” and “I feel that my colleagues and leaders are jealous of me when I achieve good results in online teaching during the pandemic”. A higher score indicates a higher level of perception of school competitive relationships. Teachers perceptions of school competitive relationships in this study demonstrated good internal consistency (Cronbach’s α = 0.89, McDonald’s ω = 0.88).

#### 3.2.2 Burnout

Based on the fact that emotional exhaustion contributes most significantly to burnout [47, 82], this study used the "Emotional Exhaustion Subscale" (8 items) of the Chinese version of the Primary and Secondary School Teachers’ Job Burnout Questionnaire (CTJBQ) [83] to assess teacher burnout at Time 2. A modified version of the CTJBQ scale was developed on the basis of the Maslach Burnout Inventory [82] to accommodate the cultural and linguistic background of mainland Chinese teachers. The CTJBQ scale includes subscales measuring emotional exhaustion, including "After a day at work, I feel exhausted" and "I feel that teaching has exhausted me emotionally and mentally." Based on a 7-point Likert-type scale, responses ranged from 1 (strongly disagree) to 7 (strongly agree). A higher score indicates a greater degree of job burnout. This study found that good internal consistency for burnout scores (Cronbach’s alpha = 0.95, McDonald’s ω = 0.95).

#### 3.2.3 Psychological distress

In order to assess psychological distress at Time 2, this research utilized the Chinese version of the Depression, Anxiety, and Stress Scale (DASS-21) developed by Chan et al. [84]. It has been demonstrated that the Chinese version of the DASS-21 scale has satisfactory psychometric properties [85, 86]. In addition, recent studies have shown that DASS-21 scores are a valid indicator of general psychological distress [87, 88]. A four-point scale was used to evaluate items on the DASS-21, with higher scores indicating more severe psychological distress. DASS-21 scores demonstrated excellent internal consistency in this study (Cronbach’s alpha = 0.96, McDonald’s alpha = 0.96).

### 3.3 Data analysis strategy

In terms of data analysis, a descriptive analysis was first conducted to analyze the background characteristics of the participants. This was followed by Pearson correlation analysis to determine the means of all variables and their correlations. As a next step, HLM 6.08 software was used to analyze the data to test the hypotheses H_1_ (H_1a_, H_1b_, H_1c_) and H_2_. HLM applies when observations in a study grouped in some way and the groups are selected randomly; therefore, it is commonly used to analyze nested data [79]. Model testing proceeded in four phases: null model, random intercepts model, means-as-outcomes model, intercepts- and slopes-as-outcomes model [89]. In this research, an intercepts-as-outcomes model was implemented, as we intended to examine the impact of school-level perceived instructional leadership on job burnout and psychological distress, rather than focusing on the moderating effect of variables. Based on this model, all the demographic variables investigated were treated as control variables except for subject of instruction, which is a category variable. Thus, more dummy variables were generated. Also, the variable for subject of instruction did not have a significant impact on the dependent variables or mediator variables and different subject teachers did not differ significantly in the means of these variables. The specific formulae for HLM are as follows:

For H_1_:

Teacher level:


$${\left(burnout\right)}_{ij}=\beta_{0j}+\beta_{1j}{\left(gender\right)}_{ij}+\beta_{2j}{\left(teaching\;experience\right)}_{ij}+\gamma_{ij}$$

School level:

$$\begin{array}{l} \beta_{0j}=\gamma_{00}+\gamma_{01}{\left(school\;type\right)}_{j}+\gamma_{02}{\left(\mathrm{neglect}\;of\;teaching\;autonomy\right)}_{j}+\gamma_{03}{\left(\mathrm{neglect}\;of\;teaching\;competence\right)}_{j}\\ \;+\gamma_{04}{\left(emphasis\;on\;competitive\;relationships\right)}_{j}+\mu_{0j}\\ \beta_{1j}=\gamma_{10},\\ \beta_{2j}=\gamma_{20}+\mu_{2j}. \end{array}$$ (1)

For H_2_:

Teacher level:

$${\left(PD\right)}_{ij}=\beta_{0j}+\beta_{1j}{\left(gender\right)}_{ij}+\beta_{2j}{\left(teaching\;experience\right)}_{ij}+\beta_{3j}{\left(burnout\right)}_{ij}+\gamma_{ij}$$

School level:

$$\begin{array}{l} \beta_{0j}=\gamma_{00}+\gamma_{01}{\left(school\;type\right)}_{j}+\mu_{0j}\\ \beta_{1j}=\gamma_{10},\\ \beta_{2j}=\gamma_{20}+\mu_{2j}\\ \beta_{3j}=\gamma_{30}+\mu_{3j}. \end{array}$$ (2)

To verify H_3_, a bootstrapping method was applied with 5000 random samples in order to test the indirect mediating effect of job burnout. Specifically, this path is labeled as a 2-1-1 model, with these three numbers representing the levels of the independent variable, mediator variable, and dependent variable. Specifically, the independent variable was at the school level (level 2) and both the mediator and dependent variable were both at the teacher level (level 1) (burnout and psychological distress). The indirect effect was tested using model 4 of Hayes’ PROCESS macro [90] by placing all variables at the teacher level, as in [91]. As a result of using the bootstrapping method, the path coefficient and confidence interval were obtained. It can be concluded that a mediation effect is established if the confidence interval does not contain 0 [92].

HLM essentially serves as an extension of regression analysis [79]. Before delving into the primary statistical analysis, we rigorously assessed key assumptions tied to regression, including linearity, multivariate normality, and the absence of autocorrelation and multicollinearity. We employed Quantile-Quantile (QQ) plots (refer to S3 and S4 Figs) to evaluate linearity and multivariate normality, with the plots closely following a straight line, indicating an approximately linear and normal distribution of residuals. For the dependent variable "burnout", the Goldfield-Quandt test (statistic = 1.08, *p* = 0.22) and the Durbin-Watson test (DW statistic = 1.93, *p* = 0.26) confirmed the absence of heteroskedasticity and significant autocorrelation, respectively. Similarly, for "psychological distress", the Goldfield-Quandt test (statistic = 0.83, *p* = 0.98) and the Durbin-Watson test (DW statistic = 2.03, *p* = 0.71) yielded consistent results. Additionally, all Variance Inflation Factor (VIF) values were below 1.7, indicating no multicollinearity issues.

## 4 Results

### 4.1 Descriptive statistics and Pearson correlations

Before presenting the results of this study, a confirmatory factor analysis (CFA) was performed using diagonally weighted least squares estimation (DWLS) in light of the fact that DWLS is more suitable to the analysis of ordinal Likert-type scales [93]. The results of the CFA were presented in the appendix (see S4 and S5 Tables). Both the model fit (CFI = 0.985, NNFI = 0.984, RMSEA = 0.037, SRMR = 0.057) and the factor loadings (larger than 0.5) demonstrated satisfactory factorial validity in this study. Furthermore, the average variance extracted (AVE) values (see Table 1) are generally greater than 0.5, indicating acceptable convergent validity.

**Table 1:** Characteristics of participants.

	*n* = 927
**Gender; *n* (%)**	
Male	172 (18.6%)
Female	755 (81.4%)
**Years of teaching experience; *n* (%)**	
Under 5 years	224 (24.2%)
6–10 years	239 (25.8%)
11–15 years	170 (18.3%)
16–20 years	92 (9.9%)
Over 20 years	202 (21.8%)
**Subject of instruction; *n* (%)**	
Chinese	332 (35.8%)
Mathematics	307 (33.1%)
English	118 (12.7%)
Natural science (physics, chemistry, biology, geography)	56 (6.1%)
Others (eg. music, art, PE)	114 (11.3%)
**School type; *n* (%)**	
primary school	282 (30.4%)
Secondary school	645 (69.6%)

Table 1 displays the demographic characteristics of the study participants, including their gender, teaching experience, subject of instruction, and school type (primary or secondary). It is estimated that 81.4% of participants are females. Regarding teaching experience, 24.2% of the participants had less than 5 years of experience, while 25.8%, 18.3%, 9.9%, and 21.8% had 6–10 years, 11–15 years, 16–20 years, and more than 20 years of experience, respectively. Among the participants, 35.8% taught Chinese, 33.1% taught mathematics, 12.7% taught English, 6.1% taught natural sciences (physics, chemistry, biology, geography), and 11.3% taught other subjects. Of the participants, 30.4% were from primary schools, and the remaining were from secondary schools.

Table 2 presents the means, standard deviations (SD), and Pearson correlation coefficients for the variables included in the study. The correlation coefficients show a significant positive association between perceived instructional leadership (perceived school neglect of teaching autonomy, school neglect of teaching competence, emphasis on competitive relationships) and burnout and psychological distress (*r* = 0.15 to 0.59).

**Table 2:** Descriptive statistics and Pearson Correlation matrix of the study variables.

	Mean (SD)	Cronbach’s α	McDonald’s ω	AVE	1	2	3	4	5
1. Perceived school neglect of teaching autonomy at Time 1	3.89 (1.66)	0.79	0.79	0.49	1.00				
2. Perceived school neglect of teaching competence at Time 1	3.89 (1.23)	0.84	0.86	0.63	0.59**	1.00			
3. Perceived school emphasis on competitive relationships at Time 1	2.39 (1.06)	0.89	0.88	0.64	0.35**	0.43**	1.00		
4. Burnout at Time 2	3.50 (1.52)	0.95	0.95	0.70	0.20**	0.23**	0.16**	1.00	
5. Psychological distress at Time 2	0.45 (0.52)	0.96	0.96	0.58	0.15**	0.21**	0.23**	0.51**	1.00

Note

*p<0.05

**p<0.01. AVE = Average Variance Extracted.

### 4.2 HLM

Table 3 presents the results of HLM analysis. The null model with job burnout and psychological distress as outcome variables yielded ICC values of 0.035 and 0.005. Despite the small ICCs, HLM was not abandoned since additional dependence on higher-level grouping can arise after including explanatory variables into the models [94]. The use of multilevel analysis is not precluded by small ICCs [10]. Therefore, we continued to use HLM for our research objectives.

**Table 3:** Results of HLM.

Parameter	Model for ICC_Burnout	Model for ICC_PD	Model for H1_Burnout	Model for H2_PD
Fixed effects				
Intercept *γ*_00_			2.17 (0.39)**	0.62 (0.10)**
*γ*_01_ School type			0.29 (0.11)*	0.08 (0.04)*
*γ*_02_ Perceived school neglect of teaching autonomy			0.38 (0.17)*	
*γ*_03_ Perceived school neglect of teaching competence			-0.02 (0.14)	
*γ*_04_ Perceived school emphasis on competitive relationships			-0.10 (0.14)	
*γ*_10_ Gender			0.36 (0.16)*	-0.14 (0.05)**
*γ*_20_ Teaching experience			0.06 (0.03)	-0.02 (0.01)
*γ*_30_ Burnout				0.18 (0.01)**
Random effects				
*σ* ^2^	2.23	0.2684	2.21	0.184
*τ*_00_	0.08	0.0013	0.005	0.039
ICC	0.035	0.0048		

PD = Psychological Distress, ICC = Intra-class correlation

*p<0.05

**p<0.01.

The results of the intercepts-as-outcomes model, displayed in Eqs (1) and (2), reveal that, after controlling for relevant variables, perceived school neglect of teaching autonomy has a significant positive impact on teachers’ job burnout (*β* = 0.38, *SE* = 0.17, *p* = 0.02), which supports H_1a_. However, perceived school neglect of teaching competence and emphasis on competitive relationships did not significantly impact burnout negatively, indicating that H_1b_ and H_1c_ were not supported. Additionally, the model shows that job burnout significantly and positively impacted psychological distress (*β* = 0.18, *SE* = 0.01, *p*<0.01), supporting H_2_.

To test the third hypothesis, the mediating effect of job burnout between perceived school neglect of teaching autonomy and teachers’ psychological distress was examined based on the results of the first hypothesis. The bootstrapping method was applied with 5000 random samples, and the indirect effect was found to be significant [indirect effect = 0.046, 95% CI (0.031, 0.061)], which supports the proposed model wherein perceived school neglecting: teaching autonomy had a significant indirect effect on teachers’ psychological distress through job burnout. Therefore, it can be concluded that perceived school neglecting: teaching autonomy has a significant impact not only on teachers’ job burnout but also on their psychological distress, highlighting the importance of addressing this issue in schools.

## 5 Discussion

The educational landscape has been profoundly affected by the COVID-19 pandemic, with the closure of schools presenting a myriad of challenges for educators. A plethora of studies have underscored the multifaceted challenges teachers faced, ranging from the rapid adaptation to novel teaching technologies [95] to an escalation in workload [9, 96]. Furthermore, a palpable lack of administrative support [10, 28, 32] has exacerbated the psychological distress experienced by educators. This research augments the existing body of knowledge by elucidating the ramifications of instructional leadership that overlooks the essence of teaching autonomy. Such neglect has been identified as a salient precursor to psychological distress, with burnout serving as a mediating factor. Notably, the study did not discern any significant effects stemming from the perceived neglect of teaching competence or the emphasis on competitive relationships within educational settings.

A pivotal revelation of this investigation is the detrimental impact of perceived institutional disregard for teaching autonomy during school closures. This adverse effect manifested prominently in the form of burnout and persisted even as educators transitioned back to traditional, in-person teaching modalities. This aligns with prior research which posits that diminished autonomy can be a catalyst for protracted burnout [27, 36, 60, 61]. Conversely, some studies [36, 97] have championed the protective role of perceived autonomy against burnout, particularly during the pandemic. These studies have enumerated several avenues to bolster teacher autonomy, encompassing flexibility in curriculum delivery, platform selection, and scheduling. Empirical evidence has consistently shown a positive correlation between teacher autonomy and pivotal outcomes such as motivation, instructional quality [98], empowerment [99] and job satisfaction [100], while inversely correlating with burnout [62]. The significance of autonomy in pedagogical settings cannot be overstated, especially given its pivotal role in teacher retention [100]. The deprivation of such autonomy, particularly in online pedagogical settings, can precipitate a cascade of negative outcomes, including diminished motivation, dissatisfaction, and pronounced burnout [62]. It’s noteworthy that the autonomy under scrutiny pertains to the latitude teachers had during online instruction, encompassing their discretion in pedagogical methodologies. The enduring impact of this neglect on educators’ mental well-being resonates with findings from Besser et al. [8] and Wakui et al. [101].

Contrastingly, this study’s findings diverge from the anticipated outcomes regarding the neglect of teaching competence and the emphasis on competitive relationships among educators. Such factors did not emerge as significant contributors to burnout. This observation is buttressed by findings from Huang et al. [102] and Yang and Huang [103], which highlight the plethora of resources available to educators during the pandemic, enabling continuous pedagogical skill enhancement. Consequently, it can be inferred that perceived school neglect of teaching competence might not be a salient determinant of burnout. Moreover, while competitive relationships can undoubtedly engender a less collegial environment, the virtual nature of instruction during the pandemic might have attenuated the impact of such competition on burnout. However, as educational institutions gravitate back to traditional teaching modalities, fostering a collaborative ethos among educators, underscored by mutual support and feedback, is paramount. This collaborative approach, coupled with the evident significance of autonomy, is pivotal for the holistic well-being of educators [104].

Further buttressing the findings of this study is the established linkage between educators’ burnout and psychological distress [38, 39, 71, 72]. Burnout, typified by sustained negative affect related to pedagogical duties, can culminate in enduring psychological distress among educators [105]. This study’s findings also corroborate the mediating role of burnout between the perceived neglect of teaching autonomy and psychological distress, aligning with the conceptualization of burnout as a strain in SSO models [42, 43, 74, 75]. Specifically, the study spotlighted the neglect of teaching autonomy by instructional leadership during school closures as a prominent stressor, culminating in protracted burnout and psychological distress.

Furthermore, the results derived from hierarchical linear modeling (HLM) underscored that perceived instructional leadership (perceived school neglect of teaching autonomy and competence, and emphasis on competitive relationships) did not have a direct bearing on psychological distress. Thus, this investigation substantiates the mediating role of burnout between perceived instructional leadership and educators’ psychological distress, aligning seamlessly with the SSO model.

Despite the valuable insights this study offers, there are several limitations to consider. Firstly, our sample was not randomly selected, which might constrain the generalizability of the findings to all middle and high school teachers in mainland China. Moreover, we did not include other teacher categories, such as kindergarten or university educators. Secondly, in order to efficiently access teachers’ perceived instructional leadership under pandemic conditions, we used a directive leadership as the special form of instructional leadership, which lead that our measurement of perceived instructional leadership is limited by epidemic. Future research would benefit from the development of a dedicated scale to assess perceived instructional leadership.

## 6 Conclusions

This study underscores the significant role that instructional leadership can play as a stressor for teachers over the long term in the pandemic, especially when it overlooks teaching autonomy. The findings indicate that when teachers perceive instructional leadership as neglecting their autonomy, it can have a profound and lasting impact on their job burnout. This, in turn, can detrimentally affect their mental well-being.

While strategies such as bolstering teacher resilience and ensuring more robust support from colleagues and managers are essential, our study also emphasizes the importance of enhancing teaching autonomy. Schools should prioritize giving teachers more ownership over their teaching methods, facilitated by sustainable leadership practices that emphasize life-long learning. Given the intricate nature of teaching, sustainability in the profession undoubtedly requires the autonomy that allows teachers to adaptively address students’ needs. This is especially true considering the challenges posed by the pandemic on teachers’ motivation and job satisfaction. As schools transition back to in-person teaching in the post-pandemic era, it becomes imperative to respect teachers’ pedagogical choices, grant them increased autonomy in the classroom, and nurture their self-efficacy and innovative capabilities. Such measures are crucial for the long-term mental health and overall well-being of teachers.

## Supporting information

S1 Checklist STROBE-checklist. (PDF)

S1 Fig The result of optimal design. (TIF)

S2 Fig The corresponding relationship between perceived instructional leadership and PNTSIOT. (TIF)

S3 Fig Q-Q Plot of residuals as burnout dependent variable. (TIF)

S4 Fig Q-Q Plot of residuals as psychological distress dependent variable. (TIF)

S1 Table Items of psychological need thwarting of online teaching scale. (PDF)

S2 Table Items of emotional exhaustion subscale. (PDF)

S3 Table Items of DASS-21. (PDF)

S4 Table Model fit. (PDF)

S5 Table Factor loadings of CFA. (PDF)

S1 File Data source. (SAV)

## References

[1] Pokhrel S, Chhetri R. A literature review on impact of COVID-19 pandemic on teaching and learning. Higher Education for the Future. 2021; 8(1): 133–141. doi: 10.1177/2347631120983481

[2] Chi X, Liang K, Chen ST, Huang Q, Huang L, Yu Q, et al. Mental health problems among Chinese adolescents during the COVID-19: The importance of nutrition and physical activity. International Journal of Clinical and Health Psychology. 2021; 21(3): 100218. doi: 10.1016/j.ijchp.2020.100218 . 33391373 PMC7759093

[3] Ye JH, Wu YT, Wu YF, Chen MY, Nong W, Lee YS. A study on construction and validation of pathways to the sustainable well-being of Chinese vocational students in the post-pandemic era. Current Psychology. 2024; 43: 7511–7525. doi: 10.1007/s12144-023-04954-x

[4] Chen H, Ye JH. (2023). The influence of outdoor activities and campus landscape on university students’ subjective well-being during the COVID-19 pandemic. Sustainability. 2023; 15(5), 4157. doi: 10.3390/su15054157

[5] Liao C, Nong L, Wu YF, Wu YT, Ye JH. The relationships between university students’ physical activity needs, involvement, flow experience and sustainable well-being in the post-pandemic era. Sustainability, 2023;15(11), 8719. doi: 10.3390/su15118719

[6] Zhou JJ, Yuan XF, Huang HH, Li YQ, Yu HY, Chen X, et al. The prevalence and correlative factors of depression among chinese teachers during the COVID-19 outbreak. Frontiers in Psychiatry. 2021; 12: 644276. doi: 10.3389/fpsyt.2021.644276 . 34267681 PMC8275853

[7] Herman KC, Sebastian J, Reinke WM, Huang FL. Individual and school predictors of teacher stress, coping, and wellness during the COVID-19 pandemic. School psychology. 2021; 36(6): 483. doi: 10.1037/spq0000456 . 34766812

[8] Besser A, Lotem S, Zeigler-Hill V. Psychological stress and vocal symptoms among university professors in Israel: implications of the shift to online synchronous teaching during the COVID-19 pandemic. Journal of voice. 2020. doi: 10.1016/j.jvoice.2020.05.028 . 32600872 PMC7274605

[9] Petrakova A, Kanonire T, Kulikova A, Orel E. Characteristics of teacher stress during distance learning imposed by the COVID‑19 pandemic. Вопросы образования. 2021; (1 (eng)): 93–114. doi: 10.17323/1814-9545-2021-1-93-114

[10] Chen IH, Chen HP, Gamble JH, Liao XL, Chen XM, Yang YT, et al. Evaluating a cross-lagged panel model between problematic internet use and psychological distress and cross-level mediation of school administrator support on problematic internet use: The serial mediating role of psychological needs thwarting of online teaching and psychological distress. Frontiers in Public Health. 2022; 10. doi: 10.3389/fpubh.2022.987366 . 36407990 PMC9667893

[11] Ye JH, Zhang M, Yang X, Wang M. The relation between intergroup contact and subjective well-being among college students at Minzu universities: The moderating role of social support. International Journal of Environmental Research and Public Health, 2023; 20(4), 3804. doi: 10.3390/ijerph20043408 . 36834102 PMC9959239

[12] Ozamiz-Etxebarria N, Berasategi Santxo N, Idoiaga Mondragon N, Dosil Santamaría M. The psychological state of teachers during the COVID-19 crisis: The challenge of returning to face-to-face teaching. Frontiers in psychology. 2021; 11: 620718. doi: 10.3389/fpsyg.2020.620718 . 33510694 PMC7835279

[13] Li Q, Miao Y, Zeng X, Tarimo CS, Wu C, Wu J. Prevalence and factors for anxiety during the coronavirus disease 2019 (COVID-19) epidemic among the teachers in China. Journal of affective disorders. 2020; 277: 153–158. doi: 10.1016/j.jad.2020.08.017 . 32828002 PMC7425543

[14] Silva DFO, Cobucci RN, Lima SCVC, de Andrade FB. Prevalence of anxiety, depression, and stress among teachers during the COVID-19 pandemic: A PRISMA-compliant systematic review. Medicine. 2021; 100(44). doi: 10.1097/MD.0000000000027684 . 34871251 PMC8568426

[15] Kupers E, Mouw JM, Fokkens-Bruinsma M. Teaching in times of COVID-19: A mixed-method study into teachers’ teaching practices, psychological needs, stress, and well-being. Teaching and Teacher Education. 2022; 115: 103724. doi: 10.1016/j.tate.2022.103724 . 35399338 PMC8977475

[16] Yıldırım M, Solmaz F. COVID-19 burnout, COVID-19 stress and resilience: Initial psychometric properties of COVID-19 Burnout Scale. Death Studies. 2022; 46(3), 524–532. doi: 10.1080/07481187.2020.1818885 . 32915702

[17] Zewude GT, Beyene SD, Taye B, Sadouki F, Hercz M. COVID-19 stress and teachers well-being: The mediating role of sense of coherence and resilience. European Journal of Investigation in Health, Psychology and Education. 2022; 13(1): 1–22. doi: 10.3390/ejihpe13010001 . 36661751 PMC9858340

[18] Wei C, Ye JH. The impacts of work-life balance on the emotional exhaustion and well-being of college teachers in China. Healthcare, 2022; 10(11), 2234. doi: 10.3390/healthcare10112234 . 36360575 PMC9689981

[19] Ye JH, Wu YT, Wu YF, Chen MY, Ye JN. Effects of short video addiction on the motivation and well-being of Chinese vocational college students. Frontiers in Public Health, 2022; 10, 847672. doi: 10.3389/fpubh.2022.847672 . 35619803 PMC9127725

[20] De Laet H, Verhavert Y, De Martelaer K, Zinzen E, Deliens T, Van Hoof E. Impact of the COVID-19 pandemic on risk of burn-out syndrome and recovery need among secondary school teachers in Flanders: A prospective study. Frontiers in Public Health. 2022; 10. doi: 10.3389/fpubh.2022.1046435 . 36579060 PMC9792144

[21] Zadok-Gurman T, Jakobovich R, Dvash E, Zafrani K, Rolnik B, Ganz AB, et al. Effect of inquiry-based stress reduction (IBSR) intervention on well-being, resilience and burnout of teachers during the COVID-19 pandemic. International Journal of Environmental Research and Public Health. 2021; 18(7): 3689. doi: 10.3390/ijerph18073689 . 33916258 PMC8037267

[22] Klusmann U, Aldrup K, Roloff-Bruchmann J, Carstensen B, Wartenberg G, Hansen J, et al. Teachers’ emotional exhaustion during the Covid-19 pandemic: Levels, changes, and relations to pandemic-specific demands. Teaching and Teacher Education. 2023; 121: 103908. doi: 10.1016/j.tate.2022.103908 . 36247186 PMC9550665

[23] Padmanabhanunni A, Pretorius TB. Teacher burnout in the time of COVID-19: Antecedents and psychological consequences. International Journal of Environmental Research and Public Health. 2023; 20(5): 4204. doi: 10.3390/ijerph20054204 . 36901219 PMC10002371

[24] Răducu CM, Stănculescu E. Personality and socio-demographic variables in teacher burnout during the COVID-19 pandemic: a latent profile analysis. Scientific Reports. 2022; 12(1): 14272. doi: 10.1038/s41598-022-18581-2 . 35995840 PMC9395542

[25] Lin Y, Ameyaw MA, Zhang Q, Sun B, Li W. The relationship between teacher professional identity and burnout amid the pandemic: A moderated mediation model. Frontiers in Public Health. 2022; 10. doi: 10.3389/fpubh.2022.956243 . 36620242 PMC9810985

[26] Pérez-Luño A, Díez Piñol M, Dolan SL. Exploring high vs. low burnout amongst public sector educators: COVID-19 antecedents and profiles. International journal of environmental research and public health. 2022; 19(2): 780. doi: 10.3390/ijerph19020780 . 35055602 PMC8776078

[27] Stan R. Personality traits, technology-related teaching skills, and coping mechanisms as antecedents of teachers’ job-related affective well-being and burnout in compulsory and higher education online teaching settings. Frontiers in Psychology. 2022; 13. doi: 10.3389/fpsyg.2022.792642 . 35519656 PMC9062184

[28] Pressley T. Factors contributing to teacher burnout during COVID-19. Educational Researcher. 2021; 50(5), 325–327. doi: 10.3102/0013189X211004138

[29] Maslach C, Schaufeli WB, Leiter MP. Job burnout. Annual review of psychology. 2001; 52(1): 397–422.: doi: 10.1146/annurev.psych.52.1.397 11148311

[30] Thompson HG, Askelson NM, Bucklin R, Gauthreaux N, Faber E, Ball C, et al. Organization-level factors associated with burnout: Guided discussions with micropolitan public health workers during COVID-19. Journal of occupational and environmental medicine, 2023; 10–1097. doi: 10.1097/JOM.000000000000283 . 36893056 PMC10329979

[31] Mijakoski D, Cheptea D, Marca SC, Shoman Y, Caglayan C, Bugge MD, et al. Determinants of burnout among teachers: a systematic review of longitudinal studies. International Journal of Environmental Research and Public Health. 2022; 19(9): 5776. doi: 10.3390/ijerph19095776 . 35565168 PMC9104901

[32] Pressley T, Ha C. Teacher exhaustion during COVID-19: Exploring the role of administrators, self-efficacy, and anxiety. The Teacher Educator. 2022; 57(1), 61–78. doi: 10.1080/08878730.2021.1995094

[33] Lavy S. Daily dynamics of teachers’ organizational citizenship behavior: Social and emotional antecedents and outcomes. Frontiers in psychology. 2019; 10: 2863. doi: 10.3389/fpsyg.2019.02863 . 31920886 PMC6931311

[34] Alanoğlu M, Demirtaş Z. Management styles’ levels of predicting job satisfaction and burnout: intermediary role of organizational justice. Egitim ve Bilim. 2020; 45(204). doi: 10.15390/EB.2020.8810

[35] Eyal O, Roth G. Principals’ leadership and teachers’ motivation: Self‐determination theory analysis. Journal of educational administration. 2011; 49(3): 256–275. doi: 10.1108/09578231111129055

[36] Collie RJ. COVID-19 and teachers’ somatic burden, stress, and emotional exhaustion: Examining the role of principal leadership and workplace buoyancy. Aera Open. 2021; doi: 10.1177/2332858420986187

[37] Westphal A, Kalinowski E, Hoferichter CJ, Vock M. K−12 teachers’ stress and burnout during the COVID-19 pandemic: A systematic review. Frontiers in psychology, 2022; 13, 920326. doi: 10.3389/fpsyg.2022.920326 . 36118449 PMC9479001

[38] Shin H, Noh H, Jang Y, Park YM, Lee SM. A longitudinal examination of the relationship between teacher burnout and depression. Journal of Employment Counseling. 2013; 50(3): 124–137. doi: 10.1002/j.2161-1920.2013.00031.x

[39] Agyapong B, Obuobi-Donkor G, Burback L, Wei Y. Stress, burnout, anxiety and depression among teachers: a scoping review. International journal of environmental research and public health. 2022; 19(17): 10706. doi: 10.3390/ijerph191710706 . 36078422 PMC9518388

[40] Hallinger P, Wang WC, Chen CW, Liare D. Assessing instructional leadership with the principal instructional management rating scale. Cham: Springer International Publishing; 2015.

[41] Şenol H, Lesinger FY. The relationship between instructional leadership style, trust and school culture. Leadership: IntechOpen; 2018.

[42] Koeske GF, Koeske RD. A preliminary test of a stress-strain-outcome model for reconceptualizing the burnout phenomenon. Journal of Social Service Research. 1993; 17(3–4): 107–135. doi: 10.1300/J079v17n03_06

[43] Tetrick LE, Slack KJ, Da Silva N, Sinclair RR. A comparison of the stress–strain process for business owners and nonowners: Differences in job demands, emotional exhaustion, satisfaction, and social support. Journal of occupational health psychology. 2000; 5(4): 464. doi: 10.1037//1076-8998.5.4.464 . 11051529

[44] Nong L, Wu YF, Ye JH, Liao C, Wei C. The effect of leisure engagement on preschool teachers’ job stress and sustainable well-being. Frontiers in Psychology, 2022; 13, 912275. doi: 10.3389/fpsyg.2022.912275 . 35936277 PMC9353011

[45] Um MY, Harrison DF. Role stressors, burnout, mediators, and job satisfaction: A stress-strain-outcome model and an empirical test. Social Work Research. 1998; 22(2): 100–115. doi: 10.1093/swr/22.2.100

[46] Freudenberger HJ. Staff burn‐out. Journal of social issues. 1974; 30(1): 159–165. doi: 10.1111/j.1540-4560.1974.tb00706.x

[47] Maslach C, Leiter MP. New insights into burnout and health care: Strategies for improving civility and alleviating burnout. Medical teacher. 2017; 39(2): 160–163. doi: 10.1080/0142159X.2016.1248918 . 27841065

[48] Bartholomew KJ, Ntoumanis N, Cuevas R, Lonsdale C. Job pressure and ill-health in physical education teachers: The mediating role of psychological need thwarting. Teaching and Teacher Education, 2014; 37, 101–107. doi: 10.1016/j.tate.2013.10.006

[49] Murtagh L, Dawes L. Examining the role of autonomy-thwarting and autonomy-supportive leadership on teacher educators during the Covid-19 pandemic. Journal of Education for Teaching, 2022; 1–12. doi: 10.1080/02607476.2022.2151880

[50] Southworth G. Instructional leadership in schools: Reflections and empirical evidence. School leadership & management. 2002; 22(1): 73–91. doi: 10.1080/13632430220143042

[51] Alig-Mielcarek J, Hoy WK. Instructional leadership: Information Age Publishers: Greenwich, CT, USA; 2005.

[52] Atalay D, Akçıl U, Özkul AE. Effects of transformational and instructional leadership on organizational silence and attractiveness and their importance for the sustainability of educational institutions. Sustainability. 2019; 11(20): 5618. doi: 10.3390/su11205618

[53] Agasisti T, Bowers AJ, Soncin M. School principals’ leadership types and student achievement in the Italian context: Empirical results from a three-step latent class analysis. Educational Management Administration & Leadership, 2019, 47(6), 860–886. doi: 10.1177/1741143218768577

[54] Garretsen H, Stoker JI, Soudis D, Wendt H. The pandemic that shocked managers across the world: The impact of the COVID-19 crisis on leadership behavior. The Leadership Quarterly, 2022, 101630. doi: 10.1016/j.leaqua.2022.101630 35719269 PMC9189185

[55] Nong LY, Ye JH, Hong JC. The impact of empowering leadership on preschool teachers’ job well-being in the context of COVID-19: A perspective based on job demands-resources model. Frontiers in Psychology, 2022; 13, 895664. doi: 10.3389/fpsyg.2022.895664 . 35693485 PMC9186648

[56] Ryan RM, Deci EL. Intrinsic and extrinsic motivations: Classic definitions and new directions. Contemporary educational psychology. 2000; 25(1): 54–67. doi: 10.1006/ceps.1999.1020 . 10620381

[57] Deci EL, Olafsen AH, Ryan RM. Self-determination theory in work organizations: The state of a science. Annual review of organizational psychology and organizational behavior. 2017; 4: 19–43. doi: 10.1146/annurev-orgpsych-032516-113108

[58] Li J, Chan PWK, Hu Y. The effects of principals’ instructional leadership on primary school students’ academic achievement in China: Evidence from serial multiple mediating analysis. Sustainability. 2023; 15(3): 2844. doi: 10.3390/su15032844

[59] Li Y, Cai Y, Tang R. Linking instructional leadership and school support to teacher expertise: The mediating effect of teachers’ professional development agency. Sustainability. 2023; 15(4): 3440. doi: 10.3390/su15043440

[60] Skaalvik EM, Skaalvik S. Dimensions of teacher self-efficacy and relations with strain factors, perceived collective teacher efficacy, and teacher burnout. Journal of educational psychology. 2007; 99(3): 611. doi: 10.1037/0022-0663.99.3.611

[61] Javadi F. On the relationship between teacher autonomy and feeling of burnout among Iranian EFL teachers. Procedia-Social and Behavioral Sciences. 2014; 98: 770–774. doi: 10.1016/j.sbspro.2014.03.480

[62] Skaalvik EM, Skaalvik S. Does school context matter? Relations with teacher burnout and job satisfaction. Teaching and teacher education. 2009; 25(3): 518–524. doi: 10.1016/j.tate.2008.12.006

[63] Franco E, Cuevas R, Coterón J, Spray C. Work pressures stemming from school authorities and burnout among physical education teachers: the mediating role of psychological needs thwarting. Journal of Teaching in Physical Education. 2021; 41(1): 110–120. doi: 10.1123/jtpe.2020-0070

[64] Brouwers A, Evers WJ, Tomic W. Self‐efficacy in eliciting social support and burnout among secondary‐school teachers. Journal of applied social psychology. 2001; 31(7): 1474–1491. doi: 10.1111/j.1559-1816.2001.tb02683.x

[65] Liu F, Chen H, Xu J, Wen Y, Fang T. Exploring the relationships between resilience and turnover intention in Chinese high school teachers: considering the moderating role of job burnout. International Journal of Environmental Research and Public Health. 2021; 18(12): 6418. doi: 10.3390/ijerph18126418 . 34199322 PMC8296230

[66] Kim LE, Asbury K. ‘Like a rug had been pulled from under you’: The impact of COVID‐19 on teachers in England during the first six weeks of the UK lockdown. British Journal of Educational Psychology. 2020; 90(4): 1062–1083. doi: 10.1111/bjep.12381 . 32975830 PMC7537096

[67] Maslach C, Leiter MP. The truth about burnout: How organizations cause personal stress and what to do about it: John Wiley & Sons; 2008.

[68] Arvidsson I, Leo U, Larsson A, Håkansson C, Persson R, Björk J. Burnout among school teachers: quantitative and qualitative results from a follow-up study in southern Sweden. BMC public health. 2019; 19(1): 1–13. doi: 10.1186/s12889-019-6972-1 . 31142318 PMC6542045

[69] Van Droogenbroeck F, Spruyt B. Do teachers have worse mental health? Review of the existing comparative research and results from the Belgian Health Interview Survey. Teaching and Teacher Education. 2015; 51: 88–100. doi: 10.1016/j.tate.2015.06.006

[70] Pyhältö K, Pietarinen J, Haverinen K, Tikkanen L, Soini T. Teacher burnout profiles and proactive strategies. European Journal of Psychology of Education. 2021; 36(1): 219–242. doi: 10.1007/s10212-020-00465-6

[71] Ozoemena EL, Agbaje OS, Ogundu L, Ononuju AH, Umoke PCI, Iweama CN, et al. Psychological distress, burnout, and coping strategies among Nigerian primary school teachers: a school-based cross-sectional study. BMC public health. 2021; 21(1): 1–15. doi: 10.1186/s12889-021-12397-x . 34969391 PMC8719383

[72] Chen IH, Chen XM, Liao XL, Zhao KY, Wei ZH, Lin CY, et al. Evaluating the immediate and delayed effects of psychological need thwarting of online teaching on Chinese primary and middle school teachers’ psychological well-being. Frontiers in Psychology. 2022; 13. doi: 10.3389/fpsyg.2022.943449 . 36051193 PMC9424862

[73] Koeske GF, Koeske RD. Student “burnout” as a mediator of the stress-outcome relationship. Research in higher education. 1991; 32: 415–431. doi: 10.1007/BF00992184

[74] Dhir A, Yossatorn Y, Kaur P, Chen S. Online social media fatigue and psychological wellbeing—A study of compulsive use, fear of missing out, fatigue, anxiety and depression. International Journal of Information Management. 2018; 40: 141–152. doi: 10.1016/j.ijinfomgt.2018.01.012

[75] Pang H. How compulsive WeChat use and information overload affect social media fatigue and well-being during the COVID-19 pandemic? A stressor-strain-outcome perspective. Telematics and Informatics. 2021; 64: 101690. doi: 10.1016/j.tele.2021.101690 . 36567817 PMC9759653

[76] Snijders TA. Power and sample size in multilevel modeling. Encyclopedia of statistics in behavioral science. 2005; 3(157), 1573.

[77] Raudenbush SW, Liu X. Statistical power and optimal design for multisite randomized trials. Psychological methods. 2000; 5(2), 199–213. doi: 10.1037/1082-989x.5.2.199 10937329

[78] Maas CJ, Hox JJ. Sufficient sample sizes for multilevel modeling. Methodology, 2005; 1(3), 86–92. doi: 10.1027/1614-2241.1.3.86

[79] Huta V. When to use hierarchical linear modeling. The quantitative methods for psychology, 2014; 10(1), 13–28. doi: 10.20892/tqmp.10.1.p013

[80] Yi J, Chen IH, Lin CY, Li CC, Liao XL, Wei ZH, et al. The effect of primary and middle school teachers’ problematic internet use and fear of COVID-19 on Psychological Need Thwarting of Online Teaching and psychological distress. Healthcare. 2021; 9(9). doi: 10.3390/healthcare9091199 . 34574973 PMC8466317

[81] Brown RD, Hauenstein NM. Interrater agreement reconsidered: An alternative to the rwg indices. Organizational research methods. 2005; 8(2): 165–184. doi: 10.1177/1094428105275376

[82] Maslach C, Jackson SE. The measurement of experienced burnout. Journal of organizational behavior. 1981; 2(2): 99–113. doi: 10.1002/job.4030020205

[83] Wu XC, Qi YJ, Yu RR, Zang WW. Revision of Chinese primary and secondary school teachers’job burnout questionnaire. Chinese Journal of Clinical Psychology. 2016; 24(05): 856–860. doi: 10.16128/j.cnki.1005-3611.2016.05.020

[84] Chan RCK, Xu T, Huang J, Wang Y, Zhao Q, Shum DHK et al. Extending the utility of the Depression Anxiety Stress scale by examining its psychometric properties in Chinese settings. Psychiatry Research, 2012; 200(2), 879–883. doi: 10.1016/j.psychres.2012.06.041 22921506

[85] Wang K, Shi HS, Geng FL, Zou LQ, Chan R. Cross-cultural validation of the Depression Anxiety Stress Scale-21 in China. Psychological Assessment. 2015; 28(5): e88. doi: 10.1037/pas0000207 . 26619091

[86] Cao CH, Liao XL, Gamble JH, Li LL, Jiang XY, Li XD et al. Evaluating the psychometric properties of the Chinese Depression Anxiety Stress Scale for Youth (DASS-Y) and DASS-21. Child and Adolescent Psychiatry and Mental Health, 2023; 17(1), 106. doi: 10.1186/s13034-023-00655-2 37679819 PMC10486035

[87] Yeung AY, Yuliawati L, Cheung SH. A systematic review and meta‐analytic factor analysis of the Depression Anxiety Stress Scales. Clinical Psychology: Science and Practice. 2020; 27(4): 190. doi: 10.1037/h0101782

[88] Zanon C, Brenner RE, Baptista MN, Vogel DL, Rubin M, Al-Darmaki FR, et al. Examining the dimensionality, reliability, and invariance of the Depression, Anxiety, and Stress Scale–21 (DASS-21) across eight countries. Assessment. 2021; 28(6): 1531–1544. doi: 10.1177/1073191119887449 . 31916468

[89] Woltman H, Feldstain A, MacKay JC, Rocchi M. An introduction to hierarchical linear modeling. Tutorials in quantitative methods for psychology, 2012; 8(1), 52–69.

[90] Hayes AF. Introduction to mediation, moderation, and conditional process analysis: A regression-based approach: Guilford publications; 2017.

[91] Sebastian J, Allensworth E. Linking principal leadership to organizational growth and student achievement: A moderation mediation analysis. Teachers College Record. 2019; 121(9): 1–32. doi: 10.1177/01614681191210090332508373

[92] Hayes AF. PROCESS: A versatile computational tool for observed variable mediation, moderation, and conditional process modeling. University of Kansas, KS; 2012.

[93] Li CH. Confirmatory factor analysis with ordinal data: Comparing robust maximum likelihood and diagonally weighted least squares. Behavior Research Methods, 2016; 48(3), 936–949. doi: 10.3758/s13428-015-0619-7 26174714

[94] Dorman JP. The impact of student clustering on the results of statistical tests. Second international handbook of science education. 2012: 1333–1348.

[95] Zheng M, Asif M, Tufail MS, Naseer S, Khokhar SG, Chen X, et al. COVID academic pandemic: Techno stress faced by teaching staff for online academic activities. Frontiers in Psychology. 2022; 13. doi: 10.3389/fpsyg.2022.895371 . 35992455 PMC9384887

[96] Kaufman JH, Diliberti MK. Divergent and inequitable teaching and learning pathways during (and perhaps beyond) the Pandemic: key findings from the American Educator Panels Spring 2021 COVID-19 Surveys. Data Note: Insights from the American Educator Panels. Research Report. RR-A168-6. RAND Corporation. 2021.

[97] Chang ML, Gaines RE, Mosley KC. Effects of autonomy support and emotion regulation on teacher burnout in the era of the COVID-19 pandemic. Frontiers in Psychology. 2022; 13. doi: 10.3389/fpsyg.2022.846290 . 35548551 PMC9083197

[98] Robertson L, Jones MG. Chinese and US middle-school science teachers’ autonomy, motivation, and instructional practices. International Journal of Science Education, 2013; 35(9), 1454–1489. doi: 10.1080/09500693.2013.792439

[99] Pearson LC, Moomaw W. The relationship between teacher autonomy and stress, work satisfaction, empowerment, and professionalism. Educational research quarterly. 2005; 29(1): 38–54.

[100] Worth J, Van den Brande J. Teacher autonomy: How does it relate to job satisfaction and retention? National Foundation for Educational Research. 2020.

[101] Wakui N, Abe S, Shirozu S, Yamamoto Y, Yamamura M, Abe Y, et al. Causes of anxiety among teachers giving face-to-face lessons after the reopening of schools during the COVID-19 pandemic: a cross-sectional study. BMC Public Health. 2021; 21(1): 1–10. doi: 10.1186/s12889-021-11130-y . 34078343 PMC8171231

[102] Huang R, Liu D, Tlili A, Yang J, Wang H. Handbook on facilitating flexible learning during educational disruption: The Chinese experience in maintaining undisrupted learning in COVID-19 outbreak. Beijing: Smart Learning Institute of Beijing Normal University. 2020; 46.

[103] Yang B, Huang C. Turn crisis into opportunity in response to COVID-19: experience from a Chinese University and future prospects. Studies in Higher Education. 2021; 46(1): 121–132. doi: 10.1080/03075079.2020.1859687

[104] Pérez-Nebra AR, Viana BS, Lira E, Martín-Hernandez P, Gracia-Pérez ML, Gil-Lacruz M. The work design contribution to educational workers’ sustainable wellbeing and performance patterns. Frontiers in psychology. 2022; 13: 1020942. doi: 10.3389/fpsyg.2022.1020942 . 36438313 PMC9682959

[105] Burić I, Slišković A, Penezić Z. Understanding teacher well-being: a cross-lagged analysis of burnout, negative student-related emotions, psychopathological symptoms, and resilience. Educational Psychology. 2019; 39(9), 1136–1155. doi: 10.1080/01443410.2019.1577952

