# Supplementary material for: The long-term effects of perceived instructional leadership on teachers’ psychological well-being during COVID-19
Source: PLoS One. 2024 Aug 19;19(8):e0305494. doi: 10.1371/journal.pone.0305494 (PMC11332923; doi:10.1371/journal.pone.0305494)
Supplement: S1 Checklist — (PDF) [file pone.0305494.s001.pdf]

STROBE Statement—checklist of items that should be included in reports of observational studies

	Item No.	Recommendation	Page No.	Relevant text from manuscript
**Title and abstract**	1	(*a*) Indicate the study's design with a commonly used term in the title or the abstract	2	long-term, longitudinal data
		(*b*) Provide in the abstract an informative and balanced summary of what was done and what was found	2	Perceived instructional leadership (particularly perceived school neglect of teaching autonomy) at Time 1 was positively associated with burnout at Time 2, while burnout at Time 2 was positively associated with psychological distress.
**Introduction**				
Background/rationale	2	Explain the scientific background and rationale for the investigation being reported	2-4	Teacher well-being during COVID-19, Job burnout among teachers
Objectives	3	State specific objectives, including any prespecified hypotheses	5-11	Teachers，Hypothesis 1, Hypothesis 2, Hypothesis 3
**Methods**				
Study design	4	Present key elements of study design early in the paper	11	This study conducted two waves of data collection: the first wave of data was collected during the period of online teaching when campuses were closed, in order to measure teachers' perceptions of instructional leadership; the second wave of data was collected after face-to-face classes resumed in order to

				evaluate teacher burnout and psychological distress.
Setting	5	Describe the setting, locations, and relevant dates, including periods of recruitment, exposure, follow-up, and data collection	11-12	Shangrao City, Jiangxi Province, China. Time 1: mid-November 2021, Time 2: early January 2022
Participants	6	(*a*) *Cohort study*—Give the eligibility criteria, and the sources and methods of selection of participants. Describe methods of follow-up<br>*Case-control study*—Give the eligibility criteria, and the sources and methods of case ascertainment and control selection. Give the rationale for the choice of cases and controls<br>*Cross-sectional study*—Give the eligibility criteria, and the sources and methods of selection of participants	12	As participation was voluntary, the data from the online survey was collected through a hyperlink with the assistance of the local education authority. participants were asked to include their email addresses if they wished to participate in a follow-up survey.
		(*b*) *Cohort study*—For matched studies, give matching criteria and number of exposed and unexposed<br>*Case-control study*—For matched studies, give matching criteria and the number of controls per case	12	email addresses
Variables	7	Clearly define all outcomes, exposures, predictors, potential confounders, and effect modifiers. Give diagnostic criteria, if applicable	13-16	Outcome: psychological distress, burnout<br>Predictor: perceived instructional leadership<br>Control variables: gender, school type (primary school or secondary school), teaching experience
Data sources/ measurement	8*	For each variable of interest, give sources of data and details of methods of assessment (measurement). Describe comparability of assessment methods if there is more than one group	13-16	the data from the online survey was collected through a hyperlink with the assistance of the local education authority,

				Perceived instructional leadership (PNTSOT), burnout (CTJBQ), psychological distress (DASS-21)
Bias	9	Describe any efforts to address potential sources of bias	12	As participation was voluntary, participants were asked to include their email addresses if they wished to participate in a follow-up survey.
Study size	10	Explain how the study size was arrived at	12	103 schools and 927 primary and secondary school teachers.

Continued on next page

Quantitative variables	11	Explain how quantitative variables were handled in the analyses. If applicable, describe which groupings were chosen and why	-	-
Statistical methods	12	(*a*) Describe all statistical methods, including those used to control for confounding	16-18	HLM, bootstrapping method
		(*b*) Describe any methods used to examine subgroups and interactions	-	-
		(*c*) Explain how missing data were addressed	-	No missing data
		(*d*) *Cohort study*—If applicable, explain how loss to follow-up was addressed<br>*Case-control study*—If applicable, explain how matching of cases and controls was addressed<br>*Cross-sectional study*—If applicable, describe analytical methods taking account of sampling strategy	12	Voluntary
		(*e*) Describe any sensitivity analyses	-	-
**Results**				
Participants	13*	(a) Report numbers of individuals at each stage of study—eg numbers potentially eligible, examined for eligibility, confirmed eligible, included in the study, completing follow-up, and analysed	12	There were 1,642 teachers who provided their email addresses and completed the longitudinal survey. To ensure data quality, we eliminated participants whose reported age was less than 18 and whose response time to all questions was less than 150 seconds. Additionally, we decided to exclude schools with participants of less than 4, considering the issue of representativeness and the required sample size [82]. The final sample for statistical analysis included 103 schools and 927 primary and secondary school teachers.
		(b) Give reasons for non-participation at each stage	-	-
		(c) Consider use of a flow diagram	-	-

Descriptive data	14*	(a) Give characteristics of study participants (eg demographic, clinical, social) and information on exposures and potential confounders	19-20	Table 1. Characteristics of participants
		(b) Indicate number of participants with missing data for each variable of interest	-	No missing data
		(c) *Cohort study*—Summarise follow-up time (eg, average and total amount)	11	Time 1: mid-November 2021; Time 2: early January 2022
Outcome data	15*	*Cohort study*—Report numbers of outcome events or summary measures over time	20-22	Results of HLM，The results of the intercepts-as-outcomes model, displayed in equations (1) and (2), reveal that, after controlling for relevant variables, perceived school neglecting: teaching autonomy has a significant positive impact on teachers' job burnout (β=0.38, SE=0.17, p=0.02), which supports H_{1a}. Additionally, the model shows that job burnout significantly and positively impacted psychological distress, supporting H_2. The bootstrapping method was applied with 5000 random samples, and the indirect effect was found to be significant [indirect effect = 0.046, 95% CI (0.031, 0.061)], which supports the proposed model wherein perceived school neglecting: teaching autonomy had a significant indirect effect on teachers' psychological distress through job burnout.
		Case-control study—Report numbers in each exposure category, or summary measures of exposure		
		Cross-sectional study—Report numbers of outcome events or summary measures		

Main results	16	(*a*) Give unadjusted estimates and, if applicable, confounder-adjusted estimates and their precision (eg, 95% confidence interval). Make clear which confounders were adjusted for and why they were included	21	Table 3
		(*b*) Report category boundaries when continuous variables were categorized	19-20	Characteristics of participants
		(*c*) If relevant, consider translating estimates of relative risk into absolute risk for a meaningful time period	-	-

Continued on next page

Other analyses	17	Report other analyses done—eg analyses of subgroups and interactions, and sensitivity analyses	-	-
**Discussion**				
Key results	18	Summarise key results with reference to study objectives	22	This study builds on previous research by demonstrating that instructional leadership, as perceived by teachers, which neglects teaching autonomy has a significant impact on psychological distress, mediated by future burnout
Limitations	19	Discuss limitations of the study, taking into account sources of potential bias or imprecision. Discuss both direction and magnitude of any potential bias	24	Limitations
Interpretation	20	Give a cautious overall interpretation of results considering objectives, limitations, multiplicity of analyses, results from similar studies, and other relevant evidence	22-25,25	Discussion, Conclusions
Generalisability	21	Discuss the generalisability (external validity) of the study results	24	the sample was not randomly selected in China, which could limit the generalizability of the findings to all middle and high school teachers in mainland China, and other types of teachers, such as kindergarten or university teachers, were not included.
**Other information**				
Funding	22	Give the source of funding and the role of the funders for the present study and, if applicable, for the original study on which the present article is based	-	This study was financially supported by the 2021 National Social Science Foundation of China (NSSFC) “Research on Mixed Ownership Model of Vocational Education” in the form of an award (BJA210105) received by I-HC. No additional external funding was received for this study. The funder

	had no role in study design, data collection and analysis, decision to publish, or preparation of the manuscript.

*Give information separately for cases and controls in case-control studies and, if applicable, for exposed and unexposed groups in cohort and cross-sectional studies.

Note: An Explanation and Elaboration article discusses each checklist item and gives methodological background and published examples of transparent reporting. The STROBE checklist is best used in conjunction with this article (freely available on the Web sites of PLoS Medicine at http://www.plosmedicine.org/, Annals of Internal Medicine at http://www.annals.org/, and Epidemiology at http://www.epidem.com/). Information on the STROBE Initiative is available at www.strobe-statement.org.
