# Supplementary figures and images for: The long-term effects of perceived instructional leadership on teachers’ psychological well-being during COVID-19
Source: PLoS One. 2024 Aug 19;19(8):e0305494. doi: 10.1371/journal.pone.0305494 (PMC11332923; doi:10.1371/journal.pone.0305494)

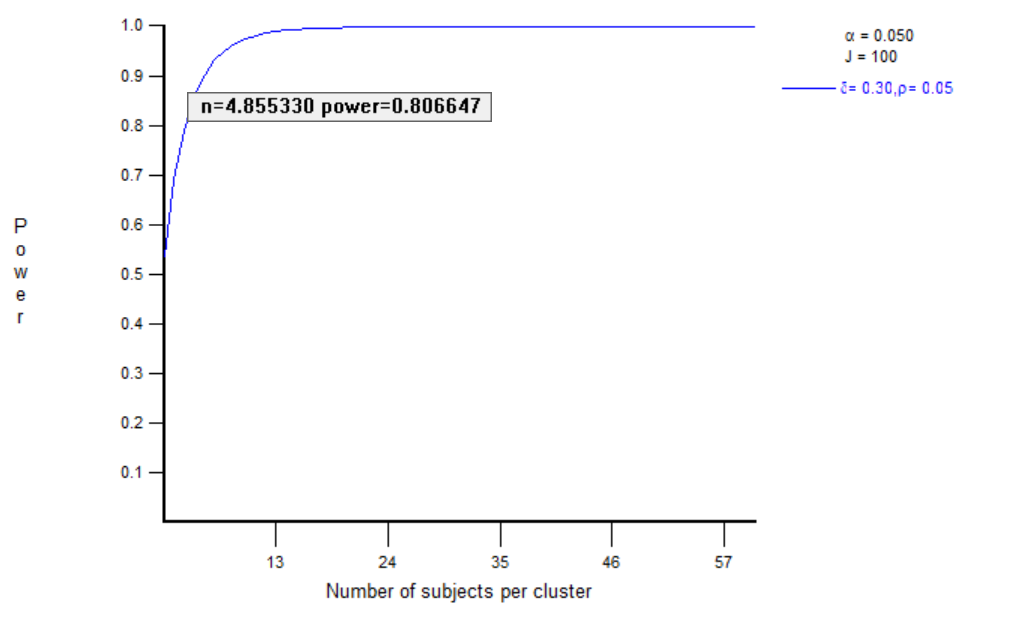

Supplement: S1 Fig — (TIF) [file pone.0305494.s002.tif]

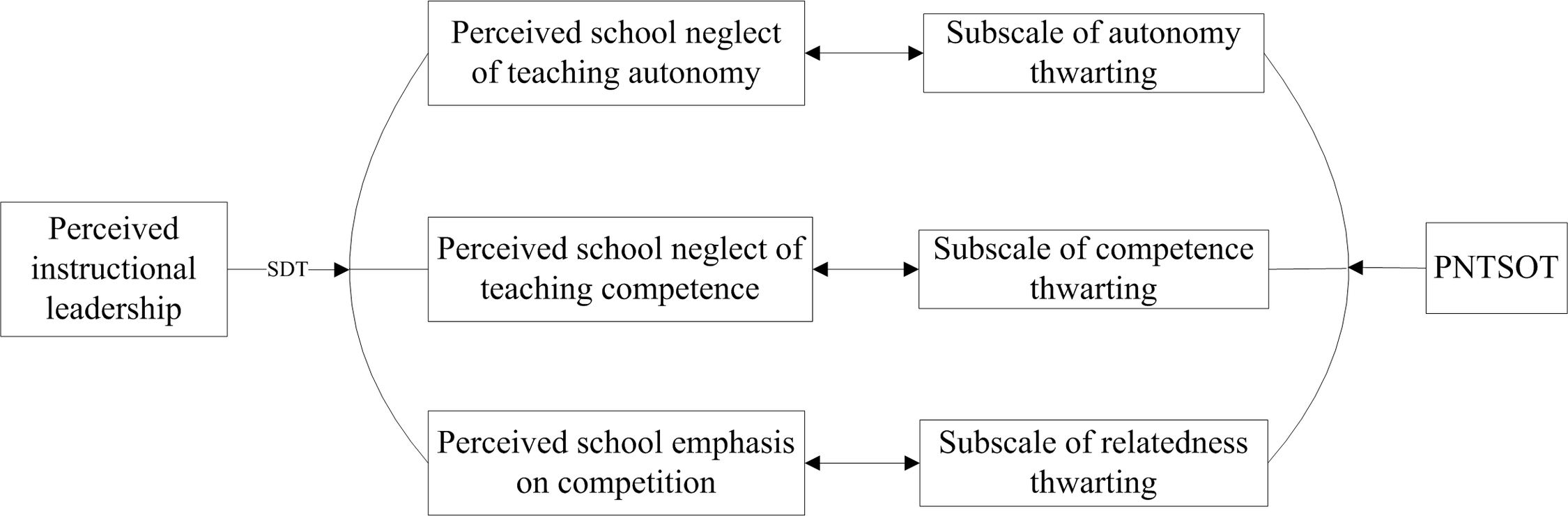

Supplement: S2 Fig — (TIF) [file pone.0305494.s003.tif]

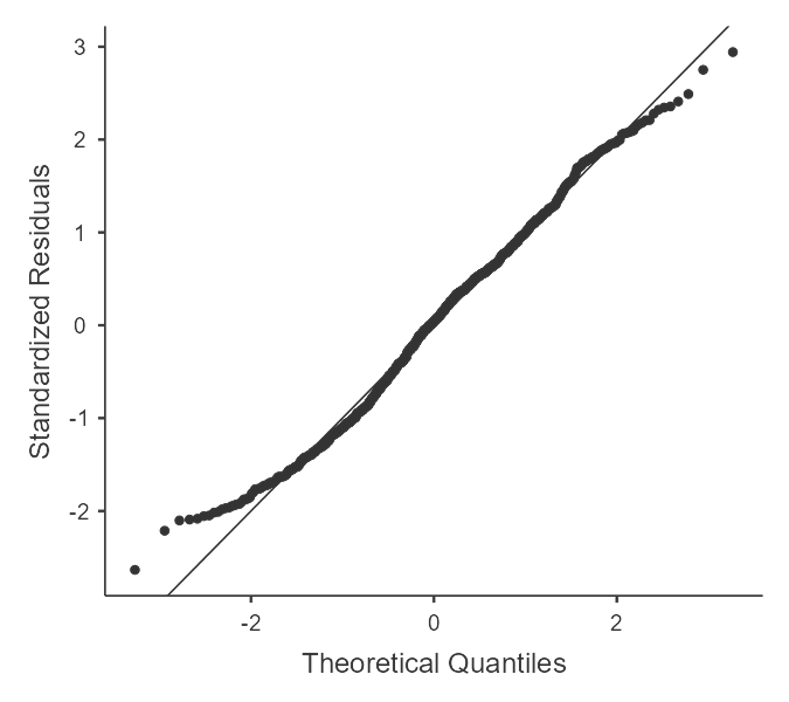

Supplement: S3 Fig — (TIF) [file pone.0305494.s004.tif]

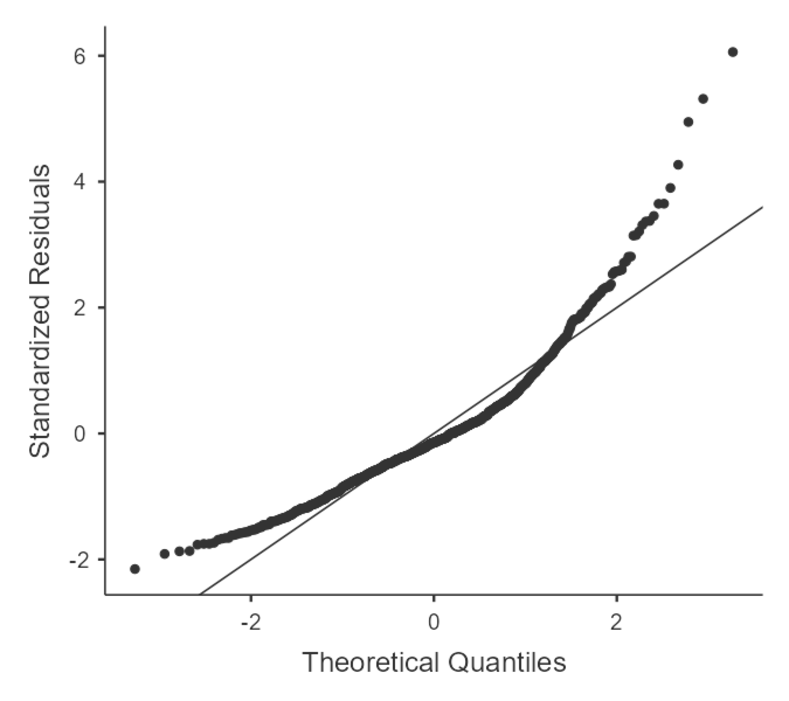

Supplement: S4 Fig — (TIF) [file pone.0305494.s005.tif]
