# Supplementary material for: The long-term effects of perceived instructional leadership on teachers’ psychological well-being during COVID-19
Source: PLoS One. 2024 Aug 19;19(8):e0305494. doi: 10.1371/journal.pone.0305494 (PMC11332923; doi:10.1371/journal.pone.0305494)
Supplement: S1 Table — (PDF) [file pone.0305494.s006.pdf]

S1 Table Items of Psychological Need Thwarting of Online teaching Scale

**Constructs**	**Items**
Perceived school neglect of teaching autonomy	1. 疫情期间的线上课程进行时，我无法自己决定想要的教学方式. In online courses during the pandemic, I cannot decide for myself how I want to teach.
	2. 疫情期间的线上教学工作进行时，我觉得有股压力会影响我的行为举止，使其符合特定规范. In online teaching work during the pandemic, I feel there is pressure that affects my behavior and requires me to comply in a certain way.
	3. 疫情期间我必需遵循某种规定的线上教学方式. I have to follow a prescribed online teaching style during the pandemic.
	4. 疫情期间，我感受到外在环境的压力，限制我必须选定特定的线上教学方式. During the pandemic, I feel pressure from the external environment that limited me in choosing a particular online teaching style.
Perceived school neglect of teaching competence	5. 疫情期间的工作环境中，有些线上教学的情况让我觉得无能为力. There are some online teaching situations that make me feel incapable in my daily work environment during the pandemic.
	6. 我有时会跟人提到疫情期间线上教学工作中让我感到无能为力的事情. I sometimes talk about the things that make me feel powerless to do my online teaching job during the pandemic.
	7. 疫情期间的线上教学工作有时会让我产生无力感. Online teaching during the pandemic sometimes makes me feel powerless.
	8. 由于环境中缺乏磨练机会，我觉得自己不能胜任线上教学的工作任务. Due to the lack of training opportunities in my environment, I feel that I am not capable of performing online teaching tasks.
Perceived school emphasis on competitive relationship	9. 疫情期间进行线上教学时，我觉得自己与其他同事及领导之间有所隔阂. I feel disconnected from other colleagues and leaders when teaching online during the pandemic.
	10. 疫情期间进行线上教学时，我无法感受到同事及领导对我的关心. I do not feel that my colleagues and leaders care about me when teaching online during the pandemic.
	11. 疫情期间的线上教学取得良好成效时，我觉得同事与领导会嫉妒我. I feel that my colleagues and leaders are jealous of me when I achieve good results in online teaching during the pandemic.
	12. 疫情期间进行线上教学时，我觉得同事与领导不喜欢我. I feel that my colleagues and leaders do not like me when I conduct online teaching during the pandemic.
