# Supplementary material for: The long-term effects of perceived instructional leadership on teachers’ psychological well-being during COVID-19
Source: PLoS One. 2024 Aug 19;19(8):e0305494. doi: 10.1371/journal.pone.0305494 (PMC11332923; doi:10.1371/journal.pone.0305494)
Supplement: S2 Table — (PDF) [file pone.0305494.s007.pdf]

S2 Table Items of Emotional Exhaustion Subscale.

Constructs	Items
Burnout	1. 我觉得自己在透支生命。 I feel emotionally drained from my work.
	2. 我觉得做老师是一份令人心力交瘁的工作。I feel teaching is really an exhausting job for me.
	3. 工作一天后，我感到筋疲力尽。After a day at work，I feel exhausted.
	4. 我觉得教学工作耗尽了我的情绪和情感。I feel that teaching has exhausted me emotionally and mentally.
	5. 从早到晚，我的脑袋里都有一根弦紧崩着，让我觉得很难受。I feel like I'm at the end of my rope.
	6. 早上起床后我会觉得很疲乏。I feel fatigued when I get up in the morning.
	7. 在工作中我有一种被掏空的感觉。I feel frustrated by my job.
	8. 我觉得我在工作中付出太多。I feel I'm working too hard on my job.
