# Supplementary material for: The long-term effects of perceived instructional leadership on teachers’ psychological well-being during COVID-19
Source: PLoS One. 2024 Aug 19;19(8):e0305494. doi: 10.1371/journal.pone.0305494 (PMC11332923; doi:10.1371/journal.pone.0305494)
Supplement: S3 Table — (PDF) [file pone.0305494.s008.pdf]

S3 Table Items of DASS-21

Constructs	Items
Psychological distress	1. 我觉得很难让自己安静下来. I found it hard to wind down.
	2. 我感到口干. I was aware of dryness of my mouth.
	3. 我好像不能再有任何愉快、舒畅的感觉. I couldn't seem to experience any positive feeling at all.
	4. 我感到呼吸困难（例如不是做运动时也感到气促或透不过气来）. I experienced breathing difficulty (e.g., excessively rapid breathing, breathlessness in the absence of physical exertion)
	5. 我感到很难自动去开始工作. I found it difficult to work up the initiative to do things.
	6. 我对事情往往作出过敏反应. I tended to overreact to situations.
	7. 我感到颤抖（例如手震）. I experienced trembling (e.g. in the hands).
	8. 我觉得自己消耗很多精神. I felt that I was using a lot of nervous energy
	9. 我忧虑一些令自己恐慌或出丑的场合. I was worried about situations in which I might panic and make a fool of myself.
	10. 我觉得自己对将来没有什么可盼望. I felt that I had nothing to look forward to.
	11. 我感到忐忑不安. I found myself getting agitated.
	12. 我感到很难放松自己. I found it difficult to relax.
	13. 我感到忧郁沮丧. I felt down-hearted and blue.
	14. 我无法容忍任何阻碍我继续工作的事情. I was intolerant of anything that kept me from getting on with what I was doing.
	15. 我感到快要恐慌了. I felt I was close to panic.
	16. 我对任何事也不能热衷. l was unable to become enthusiastic about anything.
	17. 我觉得自己不怎么配做人. l felt l wasn't worth much as a person.
	18. 我发觉自己很容易被触怒. l felt that l was rather touchy.
	19. 我察觉自己在没有明显的体力劳动时，也感到心律不正常. l was aware of the action of my heart in the absence of physical exertion (e.g., sense of heart rate increase, heart missing a beat)
	20. 我无缘无故地感到害怕. l felt scared without any good reason.
	21. 我感到生命毫无意义. I felt that life was meaningless.
