# Supplementary material for: The long-term effects of perceived instructional leadership on teachers’ psychological well-being during COVID-19
Source: PLoS One. 2024 Aug 19;19(8):e0305494. doi: 10.1371/journal.pone.0305494 (PMC11332923; doi:10.1371/journal.pone.0305494)
Supplement: S4 Table — (PDF) [file pone.0305494.s009.pdf]

S4 Table Model Fit

	Model
Standardized Root Mean Square Residual (SRMR)	0.057
Root Mean Square Error of Approximation (RMSEA)	0.037
Comparative Fit Index (CFI)	0.985
Tucker-Lewis Index (TLI)	0.984
Bentler-Bonett Non-normed Fit Index (NNFI)	0.984
Relative Noncentrality Index (RNI)	0.985
Bentler-Bonett Normed Fit Index (NFI)	0.974
Bollen's Relative Fit Index (RFI)	0.972
Bollen's Incremental Fit Index (IFI)	0.985
Parsimony Normed Fit Index (PNFI)	0.913
