# Supplementary material for: The long-term effects of perceived instructional leadership on teachers’ psychological well-being during COVID-19
Source: PLoS One. 2024 Aug 19;19(8):e0305494. doi: 10.1371/journal.pone.0305494 (PMC11332923; doi:10.1371/journal.pone.0305494)
Supplement: S5 Table — (PDF) [file pone.0305494.s010.pdf]

S5 Table Factor loadings of CFA

Construct	Items	Estimate	SE	95% Confidence Intervals		β
				Lower	Upper	
Perceived school neglect of teaching autonomy	PNTSOT1	1.000	0.0000	1.000	1.000	0.600
	PNTSOT 2	1.304	0.0549	1.197	1.412	0.749
	PNTSOT 3	1.011	0.0462	0.920	1.101	0.602
	PNTSOT 4	1.389	0.0571	1.277	1.501	0.828
Perceived school neglect of teaching competence	PNTSOT 5	1.000	0.0000	1.000	1.000	0.746
	PNTSOT 6	0.945	0.0326	0.882	1.009	0.714
	PNTSOT 7	1.044	0.0349	0.976	1.113	0.777
	PNTSOT 8	0.820	0.0283	0.765	0.875	0.737
Perceived school emphasis on competitive relationships	PNTSOT 9	1.000	0.0000	1.000	1.000	0.857
	PNTSOT10	1.058	0.0365	0.986	1.130	0.886
	PNTSOT11	0.727	0.0269	0.674	0.780	0.721
	PNTSOT 12	0.775	0.0280	0.720	0.830	0.765
Burnout	Burnout1	1.000	0.0000	1.000	1.000	0.825
	Burnout2	1.049	0.0222	1.005	1.092	0.793
	Burnout3	1.009	0.0212	0.968	1.051	0.804
	Burnout4	1.098	0.0223	1.055	1.142	0.893
	Burnout5	1.085	0.0220	1.042	1.128	0.902
	Burnout6	0.974	0.0205	0.934	1.014	0.823
	Burnout7	1.090	0.0223	1.047	1.134	0.896
	Burnout8	0.897	0.0195	0.858	0.935	0.753
Psychological Distress	DASS1	1.000	0.0000	1.000	1.000	0.636
	DASS2	1.069	0.0281	1.013	1.124	0.594
	DASS3	1.230	0.0309	1.169	1.290	0.769
	DASS4	1.098	0.0289	1.041	1.155	0.736
	DASS5	1.096	0.0284	1.041	1.152	0.748
	DASS6	1.033	0.0275	0.979	1.087	0.707
	DASS7	0.745	0.0222	0.701	0.788	0.606
	DASS8	1.410	0.0343	1.342	1.477	0.768
	DASS9	1.332	0.0329	1.268	1.397	0.807

Construct	Items	Estimate	SE	95% Confidence Intervals		β
				Lower	Upper	
	DASS10	1.387	0.0347	1.319	1.455	0.841
	DASS11	1.277	0.0320	1.214	1.340	0.856
	DASS12	1.394	0.0342	1.327	1.462	0.867
	DASS13	1.307	0.0327	1.242	1.371	0.852
	DASS14	1.085	0.0279	1.030	1.140	0.727
	DASS15	1.169	0.0304	1.109	1.229	0.828
	DASS16	1.127	0.0289	1.071	1.184	0.812
	DASS17	0.601	0.0198	0.562	0.640	0.562
	DASS18	1.253	0.0316	1.191	1.315	0.780
	DASS19	1.093	0.0283	1.037	1.148	0.772
	DASS20	1.032	0.0277	0.978	1.087	0.768
	DASS21	0.833	0.0246	0.785	0.881	0.662

Note: PNTSOTi ($1 \leq i \leq 12$) represents the ith item of Psychological Need Thwarting Scale of Online Teaching, Burnout$i$ ($1 \leq i \leq 8$) represents the ith item of Emotional Exhaustion Subscale and DASSi ($1 \leq i \leq 21$) represents the ith item of Depression, Anxiety, and Stress Scale (DASS-21).
